# A Failure to Launch: Regulatory Modes and Boredom Proneness

**DOI:** 10.3389/fpsyg.2018.01126

**Published:** 2018-07-17

**Authors:** Jhotisha Mugon, Andriy Struk, James Danckert

**Affiliations:** Department of Psychology, University of Waterloo, Waterloo, ON, Canada

**Keywords:** boredom, assessment, locomotion, goal-pursuit, self-regulation

## Abstract

Boredom is a ubiquitous human experience characterized as a state of wanting but failing to engage with the world. Individuals prone to the experience of boredom demonstrate lower levels of self-control which may be at the heart of their failures to engage in goal-directed, meaningful behaviors. Here we develop the hypothesis that distinct self-regulatory profiles, which in turn differentially influence modes of goal pursuit, are at the heart of boredom proneness. Two specific regulatory modes are addressed: Locomotion, the desire to ‘just do it,’ an action oriented mode of goal-pursuit, and Assessment, the desire to ‘do the right thing,’ an evaluative orientation toward goal pursuit. We present data from a series of seven large samples of undergraduates showing that boredom proneness is *negatively* correlated with Locomotion, as though getting on with things acts as a prophylactic against boredom. This ‘failure to launch’ that we suggest is prevalent in the highly boredom prone individual, could be due to an inability to appropriately discriminate value (i.e., everything is tarred with the same gray brush), an unwillingness to put in the required effort to engage, or simply a failure to get started. In contrast, boredom proneness was consistently *positively* correlated with the Assessment mode of self-regulation. We suggest that this association reflects a kind of rumination that hampers satisfying goal pursuit.

## Introduction

Boredom is a ubiquitous human experience associated with a raft of psychological issues including depression and anxiety (Sommers and Vodanovich, 2000; Goldberg et al., 2011), challenges in effectively engaging with rehabilitation for patients with schizophrenia and traumatic brain injury (TBI; Seel and Kreutzer, 2003; Todman, 2003; Newell et al., 2012; Branković, 2015), and impulse control issues, such as binge eating and drug abuse (Stickney and Miltenberger, 1999; Lee et al., 2007). We have characterized boredom as a negative affective state in which one is motivated to engage with the world but all attempts to do so fail (Eastwood et al., 2012; Danckert, 2013; Merrifield and Danckert, 2014). There is also considerable debate within the literature as to whether state boredom should be considered to be high or low in arousal (Abramson and Stinson, 1977; Thackray et al., 1977; Ohsuga et al., 2001; Posner et al., 2005; Mercer and Eastwood, 2010; Eastwood et al., 2012; Fahlman et al., 2013; Goetz et al., 2014; Merrifield and Danckert, 2014; Westgate and Wilson, 2017). While we do not intend to resolve that debate here, one possibility is that boredom as a state signal arises in circumstances of low arousal, whereas boredom as a trait propensity is related to failed attempts to adaptively respond to the signal – failures that may be felt as restlessness and/or agitation.

Boredom has also been characterized as a failure of goal pursuit given the inability to satisfy one’s desire to be engaged (Eastwood et al., 2012). This prompts the question of why those high in boredom proneness fail to satisfy the desire to engage in some meaningful or satisfying pursuit. There are a plethora of possibilities for this failure: an unwillingness to exert the required effort to engage with an activity, a failure to discriminate value across a range of possibilities for engagement, a failure to launch into an activity, or potentially a mismatch between one’s behavior and the optimal means of achieving the task at hand. In each instance, successful goal pursuit rests on the ability to effectively self-regulate one’s thoughts, emotions and actions (Baumeister and Vohs, 2003; Struk et al., 2016) as they relate to the task at hand, even when that task is simply *choosing* what to engage in. Indeed, trait boredom has been associated with failure to comply with treatment regimens in psychosis patients who will often quit antipsychotic therapy in order to avoid the experience of boredom, leading to detrimental consequences (Branković, 2015).

We have shown recently that boredom proneness as a trait is strongly negatively correlated with trait levels of self-control (Struk et al., 2016; Isacescu et al., 2017). That is, those with lower levels of a general measure of self-control tend to be more prone to the experience of boredom. In addition, we and others have shown that age negatively predicts boredom proneness (Vodanovich and Kass, 1990; Isacescu et al., 2017). As we get older, we are less prone to boredom. This may reflect a number of things, including the fact that with age comes increased responsibilities that may effectively limit the amount of time one has to become bored. More interestingly for our perspective, is the possibility that changes in brain development observed in late adolescence and early adulthood may underlie the reduced susceptibility to boredom. In our own work, the reduction in boredom with age remained statistically significant even when we restricted our age range to 17–22 year olds (Isacescu et al., 2017). This is precisely the age at which frontal cortices reach full maturation (Gogtay et al., 2004). In turn, both self-control and self-regulation rely heavily on the integrity of the frontal cortices (Anderson et al., 2001; Poletti, 2009; Keating, 2012).

Further support for the notion that frontal cortices are critically involved in the experience of boredom comes from a recent study in our lab of patients who had suffered traumatic brain injury (TBI; Isacescu and Danckert, 2016). Relative to age-matched controls, TBI patients had elevated levels of boredom proneness. The sine qua non of TBI is the dysexecutive syndrome, characterized by failures of inhibitory control and self-regulation (Dockree et al., 2004, 2006; O’Keeffe et al., 2007). These same behaviors – evident in increased risk taking, susceptibility to addictions of various kinds, and pathological conditions such as psychosis are also prevalent in those highly prone to boredom (Joireman et al., 2003; Mercer and Eastwood, 2010; Branković, 2015). Critical for the hypothesis developed here is the notion that optimal deployment of self-regulatory mechanisms should ward off boredom.

The measure of self-control we have made use of in the past is a broad indicator of what is likely a more nuanced set of processes (Tangney et al., 2004; Struk et al., 2016). Others have examined goal-pursuit from a variety of perspectives. One in particular, Regulatory Mode Theory (RMT; Kruglanski et al., 2000), proposes two distinct self-regulatory modes of goal-pursuit labeled Locomotion and Assessment. Locomotion emphasizes moving from one goal state to another and focuses on action implementation (i.e., “getting on with it,” or what Kruglanski and colleagues labeled the ‘just do it’ mode). In contrast, Assessment emphasizes exhaustive comparison of available alternatives (i.e., making sure to “do the right thing”; Kruglanski et al., 2000). Although orthogonal, Locomotion and Assessment represent two distinct modes in which one can pursue goals. Specific behaviors have been related to each mode based on their defining characteristics. As just one pertinent example, Pierro et al. (2011) examined the relation between regulatory mode and the tendency to procrastinate. They found that those who adopted an Assessment mode tended to procrastinate more than those adopting a Locomotion mode. Given the characteristic motivational orientation of Assessors wanting to “do the right thing,” it may be the case that prolonged evaluation of alternatives prevents, or at the very least delays the initiation of goal-pursuit. Indeed, Pierro et al. (2011) found that the relationship between Assessment and procrastination was mediated by the tendency to seek *optimal* solutions and personal fear of failure. The same association has been observed between boredom proneness and procrastination (Vodanovich and Rupp, 1999; Ferrari, 2000). In both cases, this represents a kind of ‘failure to launch’ – an inability to get started on some meaningful, satisfying task. On the other side of the coin, Pierro et al. (2011), found that Locomotors avoided procrastination in part due to a superior capacity to resist distraction – in other words, to remain focused on the task. That is, not only do Locomotors simply get on with things, they also stay on task better once a goal has been initiated. It is a well demonstrated fact that those high in boredom proneness also show impairments of sustained attention (Damrad-Frye and Laird, 1989; Cheyne et al., 2006; Carriere et al., 2008; Malkovsky et al., 2012; Hunter and Eastwood, 2016; see Eastwood et al., 2012 for review). That is, highly boredom prone individuals struggle to stay on task.

We initially explored the relationship between RMT and boredom proneness using the traditional format of the Boredom Proneness Scale (BPS) developed by Farmer and Sundberg (1986). This scale has had a checkered history in terms of determining the underlying factor structure (Ahmed, 1990; Vodanovich and Kass, 1990; Vodanovich et al., 2005). That is, a consistent factor structure has proven elusive. In our initial work on RMT we utilized the simplest two-factor structure which divides the BPS into items assessing the need for external versus internal stimulation (Vodanovich et al., 2005). It should be noted that even this structure has been called into question (Melton and Schulenberg, 2009; Struk et al., 2017). Nonetheless, we initially showed that a Locomotion regulatory mode functioned as a significant *negative* predictor of the need for internal stimulation (Struk et al., 2016). To address the short-comings of the BPS we created a short version of the scale in which reverse-scored items were reworded and items with low discriminatory value were discarded (Struk et al., 2017). This led to a single factor scale that we suggest assesses an individual’s need to be engaged with the world. While this concords with our theoretical viewpoint that boredom is a unitary construct characterized by restlessness brought on by a hindered desire to engage, it also demands a reassessment of the relations between boredom proneness and the Locomotion and Assessment self-regulatory profiles.

Here we present a theoretical account of the relationship between boredom proneness and the regulatory modes of Locomotion and Assessment and present some data to support our hypotheses. If boredom is a failure of goal pursuit, one would expect a negative relationship between the Locomotion mode and boredom proneness. This tendency toward a failure to launch into an action or goal might be the result of bored individuals wanting to make sure they ‘do the right thing,’ or at least choose the optimal path to engagement. If this is the case, one would expect a positive relationship between the Assessment mode and boredom proneness.

## Methods and Results

### Participants

Our samples were recruited from the winter, spring and fall terms of 2015, 2016 and from winter 2017 to participate in an online survey using the University of Waterloo’s Research Experience Group in which undergraduate students participate in studies for course credit. Our overall sample consisted of 12,950 students (females: 9053; *M age:* 20.33; *SD* = 3.31). All participants gave informed consent prior to completing the questionnaires. This study was approved by the University of Waterloo’s Office of Research Ethics.

### Self-Report Measures

#### Shortened Boredom Proneness Scale (SBPS; Struk et al., 2017)

The SBPS is an 8-item questionnaire that assesses one’s propensity to experience boredom. It includes items such as “I often find myself at ‘loose ends’ not knowing what to do” measured on a 7-point Likert scale ranging from 1 “Strongly disagree” to 7 “Strongly agree.” A high score on this scale reflects a high propensity to be bored. Struk et al. (2017) report an internal consistency of 0.88.

#### Regulatory Mode Questionnaire (RMQ; Kruglanski et al., 2000)

The RMQ is a 24-item questionnaire that assesses individual differences in regulatory mode. The questionnaire consists of 12 Locomotion items (e.g., “I enjoy actively doing things, more than just watching and observing”) and 12 Assessment items (e.g., “I often critique work done by myself and others”) rated on a 6-point Likert scale ranging from 1 “Strongly disagree” to 6 “Strongly agree.” High scores on each scale reflect a greater propensity for one to endorse a Locomotion and Assessment mode of goal pursuit. Kruglanski et al. (2000) report an internal consistency of 0.82 and 0.78, and a test-retest reliability of 0.77 and 0.73 for the Locomotion and Assessment scales respectively.

## Results

Test of normality indicate all variables across all samples were not normally distributed. Therefore, to test whether there was a relationship between SBPS and RMT, we conducted Spearman correlations. Descriptive statistics and correlations with SBPS are presented in **Table 1**. The mean age of our sample was 20.33 years (*SD* = 3.13). We conducted a Spearman correlation between age and boredom proneness and found that age negatively predicted boredom proneness [rho = -0.052, *p* < 0.00001].

**Table 1:** Descriptive statistics and correlations with SBPS.

Term	*N*	SBPS mean (SD)	Assessment mean (SD) Correlation^∗^	Locomotion mean (SD) Correlation^∗^	RM difference mean (SD) Correlation^∗^
W15	2127	3.23 (1.14)	3.93 (0.56)	3.90 (0.56)	0.03 (0.7)
			*r* = 0.15	*r* = -0.35	*r* = 0.40
S15	790	3.30 (1.23)	3.88 (0.69)	3.86 (0.75)	0.02 (0.72)
			*r* = 0.08	*r* = -0.35	*r* = 0.39
F15	2487	3.21 (1.61)	3.95 (0.57)	3.93 (0.57)	0.03 (0.72)
			*r* = 0.19	*r* = -0.39	*r* = 0.47
W16	1727	3.23 (1.17)	3.94 (0.56)	3.97 (0.57)	-0.03 (0.72)
			*r* = 0.20	*r* = -0.37	*r* = 0.45
S16	927	3.36 (1.19)	3.92 (0.6)	3.88 (0.6)	0.04 (0.75)
			*r* = 0.22	*r* = -0.30	*r* = 0.41
F16	2660	3.26 (1.17)	3.92 (0.6)	3.95 (0.58)	-0.03 (0.74)
			*r* = 0.22	*r* = -0.32	*r* = 0.42
W17	2232	3.27 (1.17)	3.94 (0.6)	3.93 (0.59)	0.01 (0.74)
			*r* = 0.21	*r* = -0.35	*r* = 0.43

*P < 0.0001 for all correlations. RM Difference = Assessment – Locomotion*.

**FIGURE 1:**
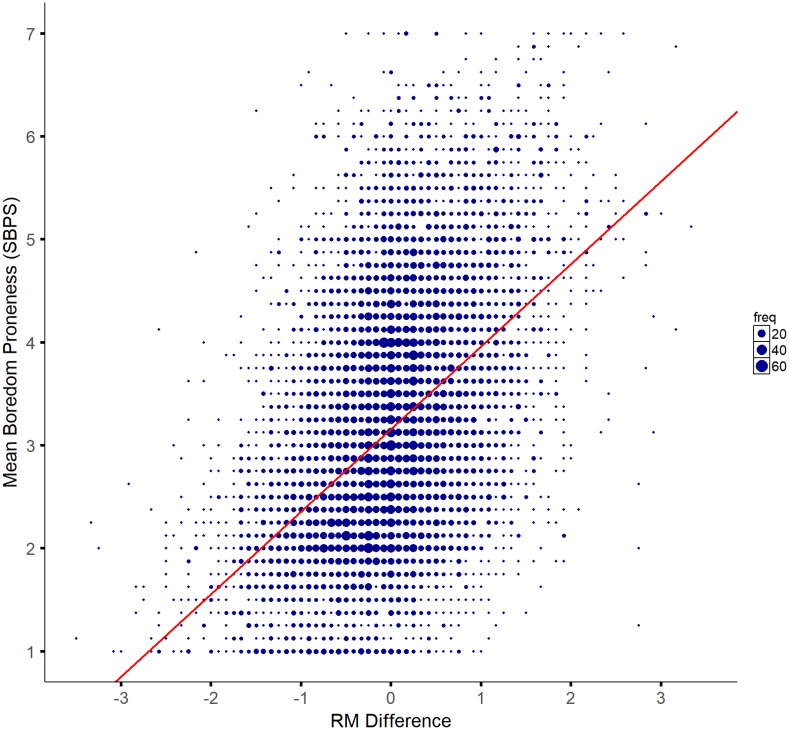
Correlation between Regulatory mode (RM) difference and boredom proneness (SBPS). Negative scores on the x-axis represent a propensity for a Locomotion mode while positive scores represent a propensity for Assessment mode. The size of data points (represented by blue dots) increase with increasing numbers of respondents with the same value (freq. legend; the smallest dot indicates 1 individual whereas the largest dot indicates 60 individuals). The red line represents a line of best fit based on the Kendall–Theil Sen Siegel nonparametric linear regression.

When boredom proneness and regulatory mode were examined, results indicated that the average scores for Boredom Proneness, Assessment and Locomotion were stable across samples (**Table 1**). We also wanted to obtain a single regulatory mode metric as an indicator of an individual’s *typical* preference for either Locomotion or Assessment. That is, for any given circumstance the same individual could choose to adopt either an Assessment or a Locomotion strategy. We reasoned that a difference score on the two scales would allow us some insight into which regulatory mode an individual normally prefers to adopt. To do this, we subtracted Locomotion scores from Assessment scores. While Assessment and Locomotion orientations can be orthogonal to each other, positive scores would indicate a general propensity for the Assessment mode, whereas negative scores would indicate a general propensity for the Locomotion mode of self-regulation. Overall, each of our samples were evenly split between demonstrating no strong preference for either Locomotion or Assessment orientations (i.e., mean scores for the samples approached zero; **Table 1**). With regards to correlations, there was a significant small to medium negative correlation between boredom proneness and Locomotion and a small yet consistently significant positive correlation between boredom proneness and Assessment across all seven samples (**Table 1**). Furthermore, there was a significant medium correlation between regulatory mode difference and boredom proneness, suggesting that as the general propensity to use the Locomotion mode increases, boredom proneness decreases (**Figure 1**).

## Discussion

The consistent negative correlation between the Locomotion and boredom proneness suggests that the tendency to simply ‘do things’ acts as a prophylactic against boredom. If one experiences little to no difficulty getting started and continuing on a given task or goal, then it seems obvious that boredom can be kept at bay. Kruglanski et al. (2000) suggest that the Locomotion system disregards how satisfying a given activity might be. Instead, it is concerned with initiating and maintaining engagement in said activity. In this light, Locomotion acts as a prophylactic against boredom because of its emphasis on movement toward a goal.

While the positive relationship between Assessment and boredom proneness is small with low variance, it is nevertheless consistent across samples suggesting that perhaps the desire “to do the right thing” is a suboptimal strategy that makes people prone to the experience of boredom. In both instances (i.e., boredom and an Assessment regulatory mode) there is a kind of rumination; while not necessarily concerned with what is the ‘right’ thing to do, the highly boredom prone individual may ruminate about exactly what will optimally satisfy their needs (as opposed to satisficing). Rumination, more generally, represents a kind of functional fixedness on the problem at hand, without any recourse to action (Nolen-Hoeksema et al., 2008). In a sense then, boredom represents a failure to launch into a satisfying activity. This notion is strengthened when looking at the preferred regulatory mode (**Figure 1**). As the tendency to adopt a Locomotion approach to goal pursuit increased, boredom proneness decreased (and vice versa). One interpretation of this would suggest that there is nothing wrong *per se* with an Assessment approach when the circumstance demands it. But a strict, inflexible adherence to this regulatory mode across a broad range of situations may make one more likely to experience boredom. Conversely, simply getting on with things likely keeps boredom at bay, perhaps regardless of the action being engaged in. This clearly warrants further research that directly manipulates the regulatory mode and task demands faced by individuals.

We are suggesting that boredom proneness and a reliance on an Assessment as opposed to a Locomotion regulatory mode, leads to rumination without action – a failure to launch. Past research has also demonstrated a positive association between rumination and depression (Mor and Winquist, 2002; Nolen-Hoeksema et al., 2008; Aldao et al., 2010). Depression in turn has consistently been positively associated with boredom proneness and Assessment and negatively associated with Locomotion (Farmer and Sundberg, 1986; Kruglanski et al., 2000; Goldberg et al., 2011; Isacescu et al., 2017). It is plausible that rumination without action itself plays a causal role in both depression and boredom. We suggest that what distinguishes boredom and depression (among many other things) is the focus of those ruminatory thoughts. For the depressed individual ruminations are typically focused inward, with negative evaluative content. For the highly boredom prone, ruminations are more commonly focused outward, critical of the boringness around them, out there in the world. It is as though the bored individual wants the world to come to them – a problem that is compounded by the fact that boredom prone individuals fail to exercise their Locomotion regulatory mode. It could also be that bored individuals view the world around them as being low in status (i.e., not engaging enough or not worth the effort; Massen et al., 2015) and thus blame the world for an apparent lack of engaging options. More research is clearly needed to evaluate these hypotheses.

Suggesting that boredom is a ‘failure to launch’ into a satisfying activity still begs the question of what causes that failure? Several possibilities present themselves to us: (1) highly boredom prone individuals may fail to appropriately discriminate value among available options; (2) they may be less willing to exert the required effort for successful engagement; (3) they may select actions that optimize goal pursuit at the expense of optimizing engagement (i.e., engendering a form of regulatory non-fit). We present these as potential avenues for further research below. One of the reasons why boredom prone individuals may fail to launch into action is because they are not able to appropriately evaluate or discern the *value* of available options. Boredom prone individuals want to be optimally engaged, but without the ability to accurately distinguish the value of different options for engagement they struggle to choose a goal they deem viable to engage with – all things seem equally viable or perhaps, equally dull. Alternatively, because boredom prone individuals are not able to discriminate value, they don’t engage in the optimally satisfying activity and thus cannot maintain engagement for long. It may be the case that boredom prone individuals need larger discrepancies in the value of options in order to be able to discriminate among them and make a choice.

Beyond the failure to accurately discriminate value, boredom prone individuals may fail to act out of diminished willingness to exert the required effort. Here, we distinguish between willingness to exert effort (do they want to exert the effort?) versus perceived effort (how much energy is required for the activity?). Thackray et al. (1977) provided evidence that those who reported experiencing boredom during a vigilance task also reported the task to *be* more effortful. That is, more energy was required for the task and participants found it difficult to sustain engagement. However, willingness to exert effort may prove critical in boredom proneness as it pertains to a failure to launch into an activity. If, as we mentioned above, boredom prone individuals want the world to come to them, then it suggests that they are less willing to put in the effort to engage in a task. As with value discrimination where boredom prone individuals need a much larger reward to first be able to discriminate whether something is ‘worth it’ to them, it could be that they also need to perceive the activity as relatively effortless in order to engage with it. As a result, boredom prone individuals limit the number of satisfying activities that they can engage with. Clearly, these two factors – the ability to discriminate value and willingness to exert effort – likely interact in complex ways. What we are putting forth here are hypotheses that may prove fruitful in understanding the mechanisms that underlie trait boredom proneness.

Finally, highly boredom prone individuals may choose means of pursuing goals that fail to *sustain* meaningful engagement. As a matter of circumstance or predisposition to “do things the right way” – as emphasized by the Assessment regulatory mode – individuals may inadvertently choose means of goal pursuit that fail to maintain engagement. This suggests that high boredom prone individuals, in addition to facing challenges of value discrimination and willingness to exert effort, may also compromise enjoyment over practicality. They fail to recognize when doing the right thing is likely to imperil engagement (e.g., taking the direct route will sink us into slow moving traffic).

In summary, we demonstrated that a Locomotion regulatory mode likely keeps boredom at bay by enabling an individual to quickly launch into and maintain engagement with a goal. Conversely, boredom proneness is in part a failure to launch into an activity that will satisfy. That failure may arise from many sources – difficulty discriminating value among available options, or a reluctance to exert the required effort for engagement represent potential candidates worth pursuing. Boredom is not a trivial experience. Elevated in a number of neurological and psychological conditions, it behooves us to develop a better theoretical account of the experience and both neurotypical and pathological responses to it. Our conceptualization of boredom as a failure to launch into a satisfying activity represents a fecund starting point for understanding this ubiquitous and problematic cognitive-affective state.

## Author Contributions

JM wrote the first draft and did the data analysis. AS and JD edited the manuscript and assisted with the data analysis.

## Conflict of Interest Statement

The authors declare that the research was conducted in the absence of any commercial or financial relationships that could be construed as a potential conflict of interest.
